# The Bone Morphogenetic Protein Pathway: The Osteoclastic Perspective

**DOI:** 10.3389/fcell.2020.586031

**Published:** 2020-10-16

**Authors:** Franziska Lademann, Lorenz C. Hofbauer, Martina Rauner

**Affiliations:** ^1^Department of Medicine III, Technische Universität Dresden, Dresden, Germany; ^2^Center for Healthy Aging, Technische Universität Dresden, Dresden, Germany

**Keywords:** bone morphogenetic proteins, osteoclasts, bone resorption, osteoblast-osteoclast coupling, bone fracture healing, recombinant BMP therapy

## Abstract

Bone health crucially relies on constant bone remodeling and bone regeneration, both tightly controlled processes requiring bone formation and bone resorption. Plenty of evidence identifies bone morphogenetic proteins (BMP) as major players in osteoblast differentiation and thus, bone formation. However, in recent past years, researchers also increasingly reported on the pivotal role of these multi-functional growth factors in osteoclast formation and activity. This review aims to summarize the current knowledge of BMP signaling within the osteoclast lineage, its role in bone resorption, and osteoblast–osteoclast coupling. Furthermore, subsequent clinical implications for recombinant BMP therapy will be discussed in view of recent preclinical and clinical studies.

## Introduction

Bone is a dynamic tissue that is maintained by continuous destruction and reformation. Within the bone remodeling cycle, osteoclasts, which derive from hematopoietic precursors, are responsible for removing old or destructed bone, while mesenchymal-derived osteoblasts synthesize and mineralize new bone matrix. The proper balance of osteoclasts and osteoblasts ensures intact bone microarchitecture, mass and function throughout life. Given that pathological osteoclast activation can lead to serious health conditions such as postmenopausal osteoporosis (Eastell et al., 2016), understanding the molecular mechanisms of osteoclast development and activity is indispensable. Latest research on the *in vivo* origin of osteoclasts identified embryonic erythro-myeloid progenitors, independent of the hematopoietic stem cell (HSC) lineage, as precursors of fetal osteoclasts crucially contributing to bone development during embryogenesis. In adult and aging mice, however, HSC-derived precursors are indispensable for postnatal osteoclast homeostasis and bone remodeling (Jacome-Galarza et al., 2019; Yahara et al., 2020). Essential cytokines involved in osteoclastogenesis are receptor activator of nuclear factor kappa-B ligand (RANKL) and macrophage colony-stimulating factor (M-CSF). M-CSF governs the survival and proliferation of osteoclast precursors by binding to its receptor c-Fms (Yoshida et al., 1990). For differentiation, RANKL is particularly important as it regulates osteoclast commitment and formation by either activating the receptor activator of nuclear factor κ B (RANK) or binding to its decoy receptor osteoprotegerin (OPG). The RANKL/RANK/OPG system controls downstream signaling such as nuclear factor κB (NF- κB), mitogen-activated protein kinase (MAPK), and c-Fos pathways as well as the master transcription factor nuclear factor of activated T-cells, cytoplasmic 1 (NFATc1) (Hofbauer et al., 2004; Takayanagi, 2007). During terminal differentiation, several osteoclast precursors fuse iteratively to become large-sized, multinuclear cells and must attach to the bone surface for bone resorption to begin (Jacome-Galarza et al., 2019). Integrins, especially integrin ανβ3, play important roles during attachment and act jointly with F-actin and actin binding proteins to form podosomes, the structural prerequisites for bone resorption. After the formation of a sealing zone, H^+^ and Cl^–^ as well as proteases such as cathepsin K are secreted into the resorption pit to dissolve the mineralized and organic structures of the underlying bone (Teitelbaum, 2000).

During this process, growth factors embedded in the bone matrix are released and help to recruit osteoblasts to the resorption area and stimulate their activity (Charles and Aliprantis, 2014). Among them, bone morphogenetic proteins (BMP) that belong to the transforming growth factor beta (TGFβ) superfamily are well-studied and vital signaling molecules controlling osteoblastogenesis and thus, bone formation. To date, 12 different BMP ligands have been identified in humans (Lowery and Rosen, 2018) and researchers accomplished to produce recombinant human BMPs (rhBMP) for research purposes, and later clinical use (Wang et al., 1990; Bessho et al., 1999). BMP signaling starts upon BMP ligand binding to a transmembranous, heterotetrameric receptor complex composed of type I BMP receptors (BMPR) (ACVR1/ALK2, BMPR1A/ALK3, BMPR1B/ALK6) and type II BMPR (BMPR2, ActR-2A, ActR-2B). Canonical BMP signaling comprises the SMAD-dependent pathway involving three types of SMADs: receptor-SMADs (R-SMADs) transducing signals, common-SMADs (Co-SMADs) supporting gene transcription activation and inhibitory-SMADs negatively regulating BMP signaling. Activated type I receptors phosphorylate R-SMADs 1, 5 and 8 enabling them to form a heterotrimeric complex with Co-SMAD4. In the nucleus, this complex acts as a transcription factor to induce the expression BMP target genes. SMAD-independent, non-canonical BMP signaling may involve MAPK, such as extracellular signal-regulated kinases (ERK) and P38, or the phosphoinositide 3-kinase (PI3K)/AKT pathway (Beederman et al., 2013; Wu et al., 2016).

## BMP Signaling in Osteoclasts: What Cell Studies and Mouse Models Tell Us

Despite the comprehensive knowledge about BMP signaling in osteoblasts, its role in osteoclast formation has long been underrated. Several studies report on the endogenous expression of several BMP ligands (BMP1, BMP2, BMP4, BMP6, BMP7), SMAD proteins (SMAD1/5, SMAD4), and BMP receptors (BMPR1A, BMPR1B, BMPR2) in osteoclasts or osteoclast-like cell lines (Anderson et al., 2000; Garimella et al., 2008; Jensen et al., 2010; Broege et al., 2013; Tasca et al., 2015, 2018).

BMP2 and BMP4, both ligands with high osteogenic potential, have also been shown to stimulate bone resorption of isolated rat osteoclasts in a dose-dependent manner (Kaneko et al., 2000). In line with this, BMP2 directly increased RANKL-mediated survival, proliferation and differentiation of murine osteoclast precursor cells (Itoh et al., 2001; Jensen et al., 2010). Interestingly, BMP2 distinctly induced canonical versus non-canonical signaling depending on the stage of osteoclast differentiation. P38 phosphorylation was increased by BMP2 only in pre-fusion osteoclasts while BMP2-mediated SMAD-activation occurred around fusion of osteoclast precursors (Broege et al., 2013). In a controversy study, RANKL and M-CSF mediated osteoclast differentiation of non-adherent human bone marrow mononuclear cells and resorption capacity were inhibited by the presence of rhBMP2 (Wan et al., 2006). BMP4 promoted osteoclast formation *in vitro* and BMP4 overexpression in osteoblasts (Col1a-Bmp4 transgenic mice) or liver (AAV8-BMP4 mice) led to elevated osteoclast numbers resulting in bone loss (Okamoto et al., 2006; Holien et al., 2018). In contrast to BMP2, BMP5 and BMP6 are less potent and enhanced osteoclast formation in a biphasic curve: at high doses (>300 mg/dl) BMP5 and BMP6 decreased and in lower doses (10–100 mg/dl) increased murine osteoclast formation (Wutzl et al., 2006). BMP7 was shown to prevent human cord blood CD14+ monocytes from differentiating into osteoclasts due to impaired persistence of important osteoclast transcription factors (Maurer et al., 2012). In contrast, a recent study demonstrated BMP7 enhanced RANKL-induced osteoclastogenesis of bone marrow derived precursors and elevated bone-resorbing activity of osteoclasts being as potent as BMP2 (Omi et al., 2019). BMP9 is able to stimulate osteoclast activity and survival through activated SMAD and ERK1/2 signaling of human cord blood monocytes-derived osteoclasts (Fong et al., 2013). All in all, the majority of studies suggest that BMP ligands increase osteoclast formation and activity while impeding osteoclast apoptosis (Table 1).

**TABLE 1:** Cell studies investigating the effects of BMP ligands on osteoclast physiology.

Cell studies			

Ligand	Species	*In vitro* osteoclast physiology	References
BMP2	Rat	⇑ Resorptive activity	Kaneko et al., 2000
	Mouse	⇑ Survival, proliferation and differentiation	Itoh et al., 2001; Jensen et al., 2010
	Mouse	⇑ Induces canonical versus non-canonical signaling depending on the stage of osteoclast differentiation	Broege et al., 2013
	Human	⇓ Differentiation and resorptive activity	Wan et al., 2006
BMP4	Rat	⇑ Resorptive activity	Kaneko et al., 2000
	Mouse	⇑ Osteoclast formation	Okamoto et al., 2006; Holien et al., 2018
BMP5	Mouse	Effect depends on concentration ⇑ Osteoclast formation with 10-100 mg/dl ⇓ Osteoclast formation with > 300 mg/dl	Wutzl et al., 2006
BMP6	Mouse	Effect depends on concentration ⇑ Osteoclast formation with 10–100 mg/dl ⇓ Osteoclast formation with > 300 mg/dl	Wutzl et al., 2006
BMP7	Human	⇓ Differentiation	Maurer et al., 2012
	Mouse	⇑ Differentiation	Omi et al., 2019
BMP9	Human	⇑ Resorptive activity and survival	Fong et al., 2013

**FIGURE 1:**
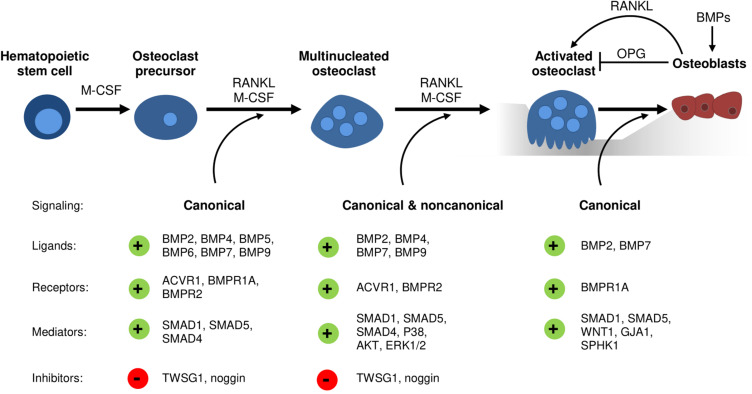
The emerging role of BMP signaling during osteoclastogenesis and osteoblast-osteoclast coupling. Conditional knockout models and cell culture studies indicate a vital role of canonical and non-canonical BMP signaling in osteoclastogenesis. Several distinct BMP ligands, BMP receptors, BMP inhibitors and downstream mediators regulate osteoclast differentiation, fusion, and resorption activity as well as osteoblast-osteoclast coupling.

With regards to BMP receptors, mechanisms regulating their expression during osteoclast development remain elusive. However, studies analyzing transgenic mouse models and thereof derived osteoclasts or precursors have implicated distinct roles of type I and type II BMP receptors in osteoclast formation and bone resorption (Table 2). Recently, ACVR1-induced SMAD-dependent BMP signaling was shown to support RANKL-induced osteoclastogenesis by activating the osteoclast master regulator NFATc1 (Omi et al., 2019). Global *Bmpr1b* knockout resulted in a transient and gender-specific osteopenia in 8-week-old male *Bmpr1b* null mice, however, *in vivo* bone turnover analysis did not detect changes in either bone formation or bone resorption (Shi et al., 2016). In contrast, *in vitro Bmpr1b*-deficient osteoclast precursors showed enhanced differentiation and survival, but decreased resorption activity (Shi et al., 2016). Deletion of *Bmpr1a* in mature osteoclasts (Bmpr1a^fl/fl^;Ctsk-Cre mice, 8-weeks-old, sex not specified) and myeloid, osteoclast precursor cells (Bmpr1a^fl/fl^;LysM-Cre mice, 8- to 10-weeks-old, male) led to trabecular bone gain due to decreased bone resorption suggesting that BMPR1A positively regulates terminal osteoclast formation and activity (Okamoto et al., 2011; Li et al., 2017). At the cellular level, osteoclast formation was impaired with *Bmpr1a*-deficiency *in vitro* (Li et al., 2017). Pharmacological blockade of type I receptors in fusion-staged osteoclasts using dorsomorphin inhibited intracellular SMAD-signaling and further osteoclast differentiation and thus, highlights the importance of canonical BMP signaling during the time of osteoclast fusion (Jensen et al., 2010; Broege et al., 2013). Conditional knockout of *BMPR2* in myeloid osteoclast precursors (BMPRII^fl/fl^;LysM-Cre mice, 12-weeks-old, male) caused trabecular bone gain due to defective osteoclast formation and activity (Broege et al., 2013). Accordingly, bone marrow derived *Bmpr2*-deficient osteoclasts showed impaired differentiation and resorptive activity indicating that BMPR2 plays an important role in osteoclastogenesis (Broege et al., 2013). Thus, both type I and type II BMP receptors are vital for proper osteoclast formation and bone resorption (Table 2), however, only during bone remodeling but not early skeletal development as bone changes are only seen in 8 to 12-week-old mice as compared to younger cohorts. Downstream of receptors, *in vitro* genetic ablation of SMAD1/5 or SMAD4 in osteoclast precursors led to the formation of fewer and smaller multinucleated osteoclasts and to a reduction of resorption pits and resorbed areas (Tasca et al., 2015). Correspondingly, mice with a conditional *Smad1/5* knockout in osteoclast precursors (Smad1^fl/fl^;Smad5^fl/fl^;c-Fms-Cre mice, 12-week-old, male) showed mild bone gain due to reduced bone resorption and stimulated bone formation (Tasca et al., 2018). In contrast, *Smad4* deletion in mature osteoclasts (Smad4^fl/fl^;Ctsk-Cre mice, 8-week-old, female) increased osteoclast formation and bone resorption leading to an osteopenic phenotype, however, caused by disrupted TGFβ signaling and independent of the BMP pathway (Morita et al., 2016). Several extracellular proteins tightly regulate BMP signaling by restricting local BMP availability and thus, may also act on the skeleton (Lowery and Rosen, 2018). Global deletion of BMP antagonist twisted gastrulation (Twsg1^–/–^ mice, 12-week-old, female) in mice induced osteopenia with increased osteoclast formation and activity caused by activated SMAD1/5 signaling in osteoclasts (Sotillo Rodriguez et al., 2009). Vice versa, *Twsg1* overexpression inhibited osteoclastogenesis (Pham et al., 2011) with BMP ligand binding being an indispensable requirement (Huntley et al., 2015). Overexpression of noggin, a prominent BMP2 and BMP4 ligand scavenger, resulted in increased bone volume due to both reduced bone formation and resorption (Okamoto et al., 2006). Accordingly, noggin restricted BMP signaling in osteoclasts and thus, osteoclast formation *in vitro* (Okamoto et al., 2006; Abe et al., 2010), however, only when administered to osteoclast precursors until day 3 of differentiation (Jensen et al., 2010). In sum, activation of BMP signaling supports osteoclastogenesis and osteoclast activity, while BMP blockade through specific inhibitors restrains proper osteoclast formation (Figure 1).

**TABLE 2:** Conditional overexpression (OE) and knockout (KO) mouse models for the analysis of BMP signaling in osteoclasts.

Mouse models					

Gene	OE/KO	Target	Bone mass	Osteoclast formation and/or activity	References
*Bmp4*	OE	Osteoblasts (Col1a promotor)	⇓	⇑	Okamoto et al.; 2006
	OE	Liver (hAAT1 promotor)	⇓	⇑	Holien et al., 2018
*Bmpr1a*	KO	Osteoclasts (Ctsk-Cre)	⇑	(⇑)	Okamoto et al., 2011
*Bmpr1a*	KO	Osteoclast precursors (LysM-Cre)	⇑	⇓	Li et al., 2017
*Bmpr1a*	KO	Osteoblasts (Col1-Cre)	⇑	⇓	Kamiya et al., 2008
*Bmpr1a*	KO	Osteocytes (Dmp1-Cre)	⇑	⇓	Kamiya et al., 2016
*Bmpr2*	KO	Osteoclast precursors (LysM-Cre)	⇑	⇓	Broege et al., 2013
*Smad1, Smad5*	KO	Osteoclasts (c-Fms-Cre)	⇑	⇓	Tasca et al., 2018
*Smad4*	KO	Osteoclasts (Ctsk-Cre)	⇓	⇑, TGFβ-mediated	Morita et al., 2016

## BMPs as Mediators Between Bone Resorption and Bone Formation

Initially, BMPs were reported to augment osteoclast development and activity solely indirectly through actions of other skeletal cells (Kanatani et al., 1995). Earlier studies demonstrate enhanced RANKL expression by bone marrow stromal cells, chondrocytes, osteoblasts, and osteocytes after stimulation with BMP ligands (Hentunen et al., 1995; Usui et al., 2008; Tachi et al., 2010; Granholm et al., 2013). Further, BMP2 is able to downregulate *Opg* mRNA levels in osteoblasts (Hofbauer et al., 1998). Eventually, an increased RANKL/OPG ratio promotes osteoclastogenesis and osteoclast function (Hofbauer et al., 2004). According to this, the BMP antagonist noggin ameliorated osteoclast formation through its effects on stromal cell/osteoblast differentiation (Abe et al., 2010). Recently, we demonstrated reduced osteoclast numbers in mice treated over 4 weeks with ALK3-Fc, a recombinant fusion protein that specifically scavenges BMPR1A/ALK3-activating BMP ligands (Lademann et al., 2020). However, due to systemic drug application it is unclear whether osteoclasts are affected in a direct or indirect manner as ALK3-Fc is reported to enhance OPG and reduce RANKL levels in serum of treated mice as well as in SaOS2 cells (Baud’huin et al., 2012). In line with this, transgenic mouse models with deleted *Bmpr1a* in either osteoblasts (Bmpr1a^fl/fl^;Col1-Cre) or osteocytes (Bmpr1a^fl/fl^;Dmp1-Cre) show enhanced bone volume not only caused by increased bone formation but also mitigated bone resorption (Kamiya et al., 2008, 2016). These effects were mediated by a decreased RANKL/OPG ratio that negatively affected osteoclast formation and function and thus, bone resorption (Kamiya et al., 2008, 2016). Vice versa, osteoclast-specific *Bmpr1a* knockout enhanced bone formation suggesting an important role of BMPR1A within osteoblast and osteoclast coupling (Okamoto et al., 2011). Furthermore, osteoblasts can be recruited by osteoclasts to sites of active bone remodeling, mediated through coupling factors such as WNT1 (Weivoda et al., 2016; Tasca et al., 2018), Gap junction alpha-1 protein (GJA1) (Tasca et al., 2018), and sphingosine kinase 1 (SPHK1) (Ryu et al., 2006; Pederson et al., 2008; Tasca et al., 2018). A recent study proposed that especially SMAD1/5-dependent signaling in osteoclasts might regulate bone formation since mRNA levels of aforementioned coupling factors were upregulated in osteoclasts with deleted SMAD1/5 (Tasca et al., 2018). In conclusion, there is manifest evidence that BMPs and their antagonists can act as mediators in osteoblast–osteoclast coupling (Figure 1) and therefore display critical determinants that dictate the rate of bone remodeling (Abe et al., 2010). Thus, time-sensitive processes such as bone healing after fracture incidences might benefit from novel therapies with targeted BMP pathway manipulation.

## Recombinant BMP Therapy and Osteoclasts: Clinical Implications

Bone has a large self-healing capacity and during fracture repair ontological events of embryonic skeletal development are recapitulated to restore the damaged skeletal tissue. This regenerative process is driven by a complex interplay of various cells, multiple growth factors and extracellular matrix, involving both anabolic and catabolic actions. Following the initial fracture, inflammatory cells invade the disrupted tissue and a hematoma is built. Subsequently, mesenchymal stem cells, the precursors of chondrocytes and osteoblasts, are recruited mainly from the periosteum and a soft, cartilaginous callus is formed by chondrocytes providing a mechanical support of the fractured area. During the following process of endochondral ossification, osteoclasts gradually resorb the soft callus matrix and highly active osteoblasts replace it by an irregularly woven and mineralized (hard) bone matrix. The final stage of remodeling involves multiple cycles of coupled bone resorption and formation as well as vascularization and reestablishes the former bone structure, strength and function (Schindeler et al., 2008; Einhorn and Gerstenfeld, 2015). Given that inadequate healing occurs in 10% of the cases, new, well-tolerated therapeutic options are needed to improve poor bone healing (Schindeler et al., 2008; Einhorn and Gerstenfeld, 2015). BMP ligands 2, 4, 6, 7, and 9 show a high osteoinductive potential as needed in case of fracture repair (Beederman et al., 2013; Wu et al., 2016). Interestingly, BMP2, BMP4, and BMP7 expression by osteoclasts is highly elevated within fracture sites of membranous fracture healing (Spector et al., 2001), In the clinics, rhBMP2 and rhBMP7 are reported to support fracture healing (Govender et al., 2002; Dumic-Cule et al., 2014), however, only in a limited subset of fractures (i.e., open tibial fractures), and in some cases are associated with side effects such as inflammation and heterotopic ossification (Fu et al., 2012; Simmonds et al., 2013; Vukicevic et al., 2014). Comprehensive reviews on fracture repair and recombinant BMPs therapy, its advantages and disadvantages can be found elsewhere (Einhorn and Gerstenfeld, 2015; Dumic-Cule et al., 2018).

As BMPs stimulate osteoclasts directly as well as indirectly via the RANKL/OPG ratio, also adverse effects of rhBMP therapy through enhanced bone resorption should be considered. In non-human primates, treatment of metaphyseal core defects with rhBMP2 delivered in an absorbable collagen sponge (ACS) resulted in transient bone resorption followed by bone formation. Animals treated with rhBMP2/ACS showed increased size of the proximal and distal core defects compared with animals treated with ACS alone. Histological analysis revealed bone resorption of the rhBMP2/ACS-treated limbs that started at 1 week and peaked at 2 weeks. Bone formation was observed at 2 weeks and was ongoing at 24 weeks (Seeherman et al., 2010). Also in patients with unstable thoracolumbar fractures, spinal fusion treatment with BMP7 as a bone graft substitute resulted in severe bone resorption as a primary event and segmental collapse (Laursen et al., 1999). This was due to a pronounced effect of high dosed rhBMP on osteoclasts and thus, enhanced substantial bone resorption at trabecular surfaces, the major part of vertebrae (Dumic-Cule et al., 2018). In retrospective analyses, initial rhBMP-stimulated bone resorption was found to be transient and followed by subsequent bone formation and repair (Fu et al., 2012; Simmonds et al., 2013). At the bone-titanium implant interface, biological response to wear particles displays the prevalent cause of aseptic loosening and osteolysis (Jacobs et al., 2001) through stimulated bone resorption (Haynes et al., 2001). A recent *in vitro* study shows that BMP2 synergizes with titanium particles to enhance RANKL-mediated osteoclast formation in osteoclast-like RAW 264.7 cells and enhanced their resorptive activity, and that at low concentrations (Sun et al., 2014). In the clinics, rhBMP2-coated devices led to early osteolysis causing implant shifts and subsequent fracture instability (Ekrol et al., 2008). To contain adverse effects of rhBMP therapy on osteoclasts while retaining the anabolic effect, several preclinical studies investigated the benefit of blocking osteoclast differentiation and activity with drugs. As such, bisphosphonates can inactivate osteoclasts by inducing apoptosis and thus, impair bone resorption (Rogers et al., 2011). Importantly, in healthy rats treated with bisphosphonates remodeling of hard fracture callus was delayed but not stopped, ensuring sufficient endochondral fracture repair (McDonald et al., 2008). In rat models with a cancellous allograft that remodels *in vivo* in a bone conduction chamber, BMPs increased the rate of bone remodeling and the volume of the remodeled graft, however, most of the newly formed bone was resorbed (Harding et al., 2008; Belfrage et al., 2011). A higher amount of newly formed bone was retained by adding bisphosphonates either locally (Belfrage et al., 2011) or systemically (Harding et al., 2008). The synergistic efficiency of combined rhBMP and bisphosphonate therapy was also shown in a critical femoral defect model (Little et al., 2005), carrier-based femoral open-fracture model (Doi et al., 2011) and femoral open-fracture models that are prone to non-union and were treated with autografts (Bosemark et al., 2013) or allografts (Mathavan et al., 2013).

Taken together, rhBMP therapy can negatively affect clinical results due to adverse effects such as simultaneously stimulation of bone resorption. Preclinical studies indicate that additionally blocking osteoclasts with bisphosphonates benefits rhBMP therapy by restricting catabolic actions while retaining anabolic effects. Still, further research is needed to fully understand the therapeutic potential and restrictions of rhBMPs in fracture repair, in particular, focusing on their effects in human osteoclasts and considering the genetic heterogeneity of patients.

## Conclusion

Bone morphogenetic proteins are multi-functional cytokines that are involved in a multitude of molecular cascades and signaling pathways. In bone remodeling, besides their essential role within bone formation, BMPs also influence osteoclast homeostasis. In this review, we show that both canonical and non-canonical BMP signaling promotes osteoclast formation and activity. In particular, BMPs support distinct steps of osteoclast differentiation and activation in a direct manner and via BMP-stimulated surrounding skeletal cells via the RANK/RANKL/OPG system. Thus, BMP signaling acts as a mediator in osteoblast-osteoclast coupling and critically affects the rate of bone remodeling. The ability of BMPs to improve poor bone healing by stimulation of osteoblasts has been reported in several clinical studies. However, rhBMP therapy can negatively affect clinical results due to adverse effects such as enhanced bone resorption. Thus, uncoupling bone formation from bone resorption through pharmacological osteoclast blockade or other approaches might be the critical step to advance rhBMP-mediated fracture repair.

## Author Contributions

FL, LH, and MR contributed to the literature research, discussion, and interpretation. FL and MR drafted the manuscript. All the authors critically read, revised, and approved the final version of the manuscript.

## Conflict of Interest

MR reports honoraria for lectures from Amgen. LH reports honoraria for advisory boards from Alexion, Amgen, Merck, Radius, Roche, Shire, and UCB to his institution and himself. The remaining author declares that the research was conducted in the absence of any commercial or financial relationships that could be construed as a potential conflict of interest.
